# Temporal Relation between Double Fortification of Wheat Flour with Iron and Folic Acid, and Markers and Prevalence of Anemia in Children

**DOI:** 10.3390/nu13062013

**Published:** 2021-06-11

**Authors:** Flinle Danielle Biemi, Vijay Ganji

**Affiliations:** 1 Department of Nutrition, Georgia State University, Atlanta, GA 30302, USA; daniellebiemi@gmail.com; 2 United Nations International Children’s Emergency Fund (UNICEF), Abidjan 04 BP 443, Côte d’Ivoire; 3 Human Nutrition Department, College of Health Sciences, QU Health, Qatar University, Doha P.O. Box 2713, Qatar

**Keywords:** children, folic acid, fortification, iron, Ivory Coast, wheat flour

## Abstract

Fortification of wheat flour with iron and folic acid became mandatory in Ivory Coast in 2007. The purpose of this study was to determine the time trend relation between mandatory double fortification of wheat flour with iron and folic acid and markers and prevalence of anemia by comparing the data between pre- and post-fortification periods in Ivory Coast children. Data were derived from the pediatric unit of the University Hospital of Treichville, Abidjan, Ivory Coast. Medical records of 467 children from 5 to 14 years old were analyzed from the years 2004 through 2010. Periods from 1 January 2004 to 31 December 2006 and 1 January 2008 to 31 December 2010 were considered as pre- and post-fortification periods, respectively. Data on hemoglobin, hematocrit, red blood cell count (RBC), mean corpuscular volume (MCV), and anemia between pre- and post-fortification periods were compared. There were no significant differences in hemoglobin, hematocrit, RBC, and prevalence of anemia between pre- and post-fortification periods. However, MCV in post-fortification period was significantly higher compared to pre-fortification period in all subjects (77.6 fL vs. 76.8 fL; *p* = 0.02) and in young girls (79.1 fL vs. 75.2 fL; *p* = 0.01). Lack of significant differences in anemia and in markers of anemia between pre- and post-fortification periods need further investigation in children of Ivory Coast.

## 1. Introduction

Anemia from micronutrient deficiencies is a major public health problem in developing countries such as Ivory Coast [1]. Anemia is also caused by other nutritional deficiencies, bacterial and viral infections, inflammation, and parasitic infestations [2]. Anemia affects nearly 2 billion people across the world [3]. In developing countries, the prevalence of anemia is estimated at 43%, while it is at 9% in developed countries [4]. Mild to moderate anemia leads to weakened immunity, reduced work capacity, reduced cognitive function, and an overall decreased quality of life. Severe anemia is considered a major cause of maternal and infant morbidity and mortality in developing countries [5]

Anemia caused by iron deficiency is the most prevalent nutritional deficiency [3]. In Ivory Coast, the prevalence of iron deficiency and iron-deficiency anemia were 41–63% and 20–39%, respectively [6]. A survey conducted prior to fortification in 900 households in 9 regions of Ivory Coast revealed that the prevalences of anemia in pre-school children and women of reproductive age (15–49 years old) were 72% and 50%, respectively [7]. Folate, another micronutrient is often deficient in the foods of people living in the developing world. Primarily, folate is present in plant-based foods. Food processing (heating, frying, milling, and baking) destroys a significant amount of folate in the food [8], which further lowers the available folate in food. Folate deficiency causes birth defects such as spina bifida, a neural tube defect, and macrocytosis, characterized by an elevated mean corpuscular volume (MCV) [9]. Folate deficiency in the women of reproductive age was 80% based on the needs assessment survey prior to food fortification in Ivory Coast. Therefore, the targeted populations for the double fortification of wheat flour with iron and folic acid were children and women of reproductive age [7]. To address micronutrient deficiencies, public health systems have developed three main strategies. These are to promote nutrition education combined with food diversification, promote iron and folic acid supplementation, and implement mandatory food fortification with iron and folic acid [10]. Among these strategies, evidence supports that food fortification is the most cost-effective, long-term approach to reduce nutrient deficiency in populations [11,12]. In various countries, iron and folate fortifications have been associated with a reduction in the prevalence of anemia in children [13,14,15,16].

In Ivory Coast, double fortification of wheat flour with iron and folic acid became mandatory in 2007 (Decree-025 was issued on 18 January 2007) with the collaboration of Non-Governmental Organizations such as the Global Alliance for Improved Nutrition and the Helen Keller International [17,18]. The double fortification of wheat flour was designed to deliver sufficient amounts of iron and folic acid to at-risk populations such as children, women of reproductive age, and pregnant women. The impact of food fortification efforts in Ivory Coast on the prevalence of anemia is under-investigated. In 2016, Rohner et al. [19] reported an improved iron and folate status and increased hemoglobin in children who had access to fortified foods in Ivory Coast. Recently, Prieto-Patron et al. [20] using the comparative risk assessment model showed that a reduction of ≈5% in iron deficiency anemia due to food fortification in Ivory Coast. No study has been conducted on a temporal association between food fortification periods (pre- and post) and markers and prevalence of anemia in children of Ivory Coast. Therefore, the objective of this study was to investigate whether children in Ivory Coast had decreased prevalence of anemia and improved markers of anemia in the post-fortification period compared to the pre-fortification period.

## 2. Methods

### 2.1. Study Setting

Ivory Coast is a west sub-Saharan African country with a tropical climate and vegetation dominated by forest and savannah. Abidjan, the economic capital of Ivory Coast, is the most populous city. The common diet of the population is composed of grains (rice, maize, and millet) and tubers (plantains, yam, and cassava). The data used in this study were derived from the medical records of the pediatric unit of the University Hospital of Treichville, one of the three main hospitals in the Abidjan area. This hospital receives patients from all parts of the country.

### 2.2. Subjects and Study Design

The design of this study was retrospective, observational on Ivorian children. Ethical approval was obtained from the hospital administration at the pediatric clinic of University Hospital of Treichville, Abidjan, prior to conducting this study (approval reference number: 60-MSLS/CHU-T/ADM-PED/KJ). As the study was retrospective in nature, we did not have contact with any participants. We considered a period before 2007 as a pre-fortification period and a period after 2007 as a post-fortification period. As the mandatory fortification commenced in January 2007, all the wheat flour would have been fortified by the end of 2007. Therefore, we determined the period beginning 2008 as the post-fortification period.

We calculated the sample size based on the Cochran’s formula (*n* = z^2^pq/e^2^; z = desired confidence level, which is 95%; *p* = estimated prevalence of anemia in the children population visiting hospital, ≈70% or 0.7; q = 1 − p, which is 1 − 0.7 = 0.3; and e = desired precision level, which is ± 5% or 0.05). Therefore, the minimum sample size needed for this study was ≈323. Had we used the adjusted Cochran’s formula for finite population (which is the case for the 5–14 years-old children attending the hospital in Ivory Coast), the minimum sample size required would have been even lower.

Retrospectively, we analyzed the medical records of 500 children, aged 5 to 14 years old. These subjects received hospital consultation during the study periods. From a total of 500 children, 250 came from the pre-fortification period and another 250 came from the post-fortification period. All 500 subjects had complete blood count measurements. Children who were hospitalized after initial consultation were considered subjects having serious medical condition. These subjects were excluded from the data analysis (*n* = 33). Thus, the final study sample contained 467 children.

### 2.3. Data Collection

Data on hemoglobin, hematocrit, red blood cell count (RBC), and MCV, and demographic characteristics (age and sex) were collected from medical records. Anemia was defined as having hemoglobin ˂11 g/dL for children under 6 years old and ˂12 g/dL for children 6–14 years old based on World Health Organization criteria [21].

### 2.4. Statistical Analysis

The Statistical Package for the Social Sciences (SPSS) version 20.0 (Chicago, IL, USA) was used for statistical analysis. A normality test was performed on the data. As the measurement variables were not normally distributed, a non-parametric test, Mann-Whitney U test was used to compare the mean difference in hemoglobin, hematocrit, RBC, and MCV between pre-fortification and post-fortification periods. The chi-squared test was performed to compare the prevalence of anemia between the pre- and post-fortification periods. The level of statistical significance was set at <0.05.

## 3. Results

Out of 467 subjects, 232 (121 boys and 111 girls) were in the pre-fortification period and 235 (123 boys and 112 girls) were in the post-fortification period. Means of hemoglobin and hematocrit by sex and age in pre- and post-fortification periods are presented in Table 1. Overall mean hemoglobin concentration was 10.9 g/dL. Hemoglobin concentration and hematocrit did not significantly differ between pre- and post-fortification periods in all subjects, all boys (both age groups), and all girls (both age groups).

Mean RBC and MCV by sex and age in pre-fortification and post-fortification periods are presented in Table 2. Overall, mean RBC did not differ significantly between pre- and post-fortification periods. In all subjects, mean MCV was significantly higher in the post-fortification period (77.6 fL in 2008–2010) compared to the pre-fortification period (76.8 fL in 2004–2006) (*p* = 0.02). However, in all boys and all girls, the MCV was not significantly different between pre- and post-fortification periods, although there was a trend (*p* = 0.07). In younger girls (5–9 years old), the MCV was significantly higher in the post-fortification period (79.1 fL in 2008–2010) compared to the pre-fortification period (75.2 fL in 2004–2006) (*p* = 0.01).

The prevalence of anemia by sex and age in pre-fortification and post-fortification periods are presented in Table 3. The overall prevalence of anemia in the entire sample was ≈77%. There was no significant difference in the prevalence of anemia between pre- and post-fortification periods in all subjects, all boys (both age groups), and all girls (both age groups).

## 4. Discussion

In this study, we reported the first data on a temporal association between double fortification of wheat flour with iron and folic acid, and anemia and makers of anemia in Ivory Coast children. We compared the differences between the pre-fortification period (2004 to 2006) and post-fortification period (2008–2010) in indices and prevalence of anemia in children aged 5–14 years old. The main finding was that we found no temporal association between pre- and post-fortification periods and indices of anemia, except MCV. Hemoglobin, hematocrit, RBC, and prevalence of anemia in children of Ivory Coast did not change from the pre-fortification to the post-fortification period. Interestingly, MCV significantly increased from pre- to post-fortification periods.

The impact of global food fortification programs on blood markers has yielded mixed results. In an Indian placebo control trial, hemoglobin concentrations did not change in schoolchildren after a community-level micronutrient fortification program [22]. A South African study also reported no improvement in hemoglobin concentration after 34 weeks of iron fortification program in schoolchildren (*n* = 361; 6–11 year old) belonging to low socioeconomic strata [23]. Similarly, a study from Brazil on pre-school-age children did not find any improvement in the prevalence of anemia from the pre-fortification period (2004) to the post-fortification period (2005–2008) although wheat flour supplied an adequate amount of iron. The authors attributed this to the low bioavailability of iron salts used to fortify wheat flour and the poor quality of diets of children [24].

However, several studies have reported the positive impact of food fortification efforts on the prevalence of anemia and the markers of anemia. Earlier, we reported an improvement in hemoglobin and hematocrit in the post- compared to the pre-folic-acid fortification period in a sample (*n* = 26,596; men = 12,670; women = 13,926) obtained from the US nationally representative sample surveys, NHANESs [25]. The effectiveness of iron fortification programs was ascertained in many countries [26,27,28,29]. A recent meta-analysis study of 10 randomized controlled trials (*n* = 3319) conducted in several countries (India, Brazil, Bangladesh, Philippines, South Africa, Sri Lanka, and Kuwait) reported that fortified wheat flour with or without other micronutrients compared to non-iron-fortified wheat flour reduced the risk of anemia by 27%. These studies used 41 to 60 mg of iron per kg of wheat flour. The length of the studies was 3 to 24 months [30]. In Venezuela, the fortification of wheat and maize flour with 50 mg/kg of iron, vitamin A, and B vitamins was associated with a significant decrease in the prevalence of anemia from 19% to 10% and iron deficiency from 37% to 16% one year later, in 307 school children aged 7 to 15 years old [28]. In a randomized controlled trial conducted in India, a wheat flour-based meal fortified with 6 mg of iron reduced the prevalences of iron deficiency anemia from 18% to 9% and iron deficiency from 62% to 21% in school children aged 6 to 15 year old (*n* = 401) [29]. Finally, in a Costa Rican national study, in which 2002 was the pre-fortification period and 2008–2009 was the post-fortification period, wheat flour fortified with iron reduced the prevalence of anemia from 19.3% to 4% and iron deficiency from 26.9% to 6.8% in pre-school children aged 1 to 7 years old [31]. In the majority of these studies, the positive effect of food fortification on the prevalence of anemia cannot be solely attributed to iron although it may have played a major role.

An interesting finding of this study was an increased MCV in the post-fortification period compared to the pre-fortification period. In our earlier study on the US population using the NHANES data, we reported a significant increase in MCV in some sub-group populations [24]. Similarly, Hirsch et al. [32] found an elevated MCV in the post-folic acid fortification period compared to the pre-fortification period. They concluded that increased MCV in the post-folic-acid fortification period is due to increased vitamin B12 deficiency. In contrast, using hospital data in the Chicago area, we reported a significant decrease in MCV from the pre- to post-folic-acid fortification period (94.4 fL to 88.6 fL) [33]. In the case of Ivorian children, whether the increased MCV is due to the increased prevalence of vitamin B12 deficiency needs further investigation.

In this current study, the lack of improvement in markers and prevalence of anemia from pre- to post-fortification periods in children of Ivory Coast could be attributed to several factors. First, the choice of food vehicle for the fortification program could partly explain these findings. Research has identified that wheat flour was the best vehicle for the iron fortification program because the bioavailability of iron added to wheat flour is greater than that of added to other staple foods such as maize and rice [34]. Nevertheless, the selection of wheat flour as a vehicle for mandatory double fortification may not be a suitable choice for the Ivorian population. According to FAO/WHO recommendations, the food to be fortified must be consumed in sufficient amounts by nearly all people in the target population [35]. In countries with higher wheat consumption such as South America where the intake was estimated to be more than 100 g/capita/day, wheat flour is the best vehicle for food fortification [36]. In Ivory Coast, however, only 70 g/capita/day of wheat flour is available versus 269 g/capita/day of available rice [37]. The consumption of wheat flour is therefore too low to deliver sufficient amount of micronutrients through fortification. In this case, rice would be a better vehicle for food fortification. Efforts on iron fortification of rice have begun in countries such as Philippines, Papua New Guinea, and Indonesia but no large-scale programs have been implemented [34]. In Philippines, evidence supports the effectiveness of iron-fortified rice in reducing anemia in preschool children [38]. Further research is therefore, needed to develop a rice fortification program to ensure an efficient alternative to wheat flour.

Another plausible explanation for the lack of improvement in anemia and in markers of anemia in the post-fortification period compared to the pre-fortification period is the inadequate fortification of wheat flour in Ivory Coast. The mandatory fortification amounts are 60 mg of elemental iron and 1.5 mg of synthetic folic acid per kg of wheat flour [39]. These fortificants have higher bioavailability compared to other forms. Based on a study in Ivory Coast by Rohner et al. [39] reported that overall only 32% of wheat flour was adequately fortified, although all flour samples contained some iron. The under-fortification of wheat flour was much higher (53%) in the rural areas compared to the urban areas such as Abidjan (7%) [19]. Under-fortification has also been reported in some African nations. For example, in Tanzania 83.3% and 15% of wheat flour samples were adequately fortified with iron and folic acid, respectively [40]. It is important to monitor the fortification levels on a continuous basis and to consider other staple foods for expansion of fortification efforts.

In children of Ivory Coast, iron and folate deficiencies are not the only causes of anemia. Infectious diseases, intestinal parasitic diseases, and congenital hemoglobinopathies (sickle-cell and thalassemias) are other causes of anemia [41,42,43]. As inflammation impairs iron absorption and utilization, the efficacy of iron fortification may be jeopardized in the population of sub-Saharan Africa where the infection rates are very high [18]. Recently Righetti et al. [41] reported a prevalence of helminth infection of 53% in school-age children, although there was a regional variability within Ivory Coast. In this context, evidence suggests that an active helminths control must be coupled with efforts to control iron deficiency anemia [44]. In addition, malaria is a health burden for children living in Ivory Coast. A recent study on Ivorian schoolchildren reported a *Plasmodium* prevalence of 74% [41]. Therefore, the high prevalence of inflammatory conditions associated with parasitic and malarial infection could partly explain the inefficacy of fortification efforts in Ivorian children.

The results of the present study should be viewed in light of some limitations. Due to observational study, the causality should not be assumed. As the study was conducted in one hospital in the capital city of Ivory Coast, these results cannot be applied to the Ivorian children at large. Overall, mean hemoglobin concentration was low (10.9 g/dL) and the prevalence of anemia was high (≈77%) in both pre- and post-fortification periods. This might have affected the results of this study. To what extent the health status affected the results is not known because in both fortification periods, the sample originated from the same hospital with a largely similar health status of participants. Due to a lack of dietary intake data, we were unable to assess the intakes of iron and folic acid. There is also a possibility that if the sample size was larger, we might have seen a significant difference in anemia between pre-and post-fortification periods. We evaluated the post-fortification data for only 2 years. Further studies are needed to assess the effectiveness of the wheat fortification program for a longer duration in the post-fortification period. Future research should explore the feasibility of fortification of rice and rice products and various condiments with anti-anemic micronutrients in Ivory Coast.

## Figures and Tables

**Table 1:** Hemoglobin and hematocrit by sex and age in the retrospective study on the children of Ivory Coast: comparison of data between pre-fortification period (2004–2006) and post-fortification period (2008–2010) ^1^.

	Hemoglobin, g/dL			Hematocrit, %		
	Pre ^2^	Post ^2^	*p*-Value ^2^ (Pre vs. Post)	Pre ^2^	Post ^2^	*p*-Value ^2^ (Pre vs. Post)
All subjects	10.9 ± 1.5	10.9 ± 1.4	0.9	32.7 ± 4.5	32.9 ± 4.3	0.9
All boys Age	10.7 ± 1.4	10.7 ± 1.3	0.9	32 ± 4.2	32.2 ± 4.3	0.9
5–9, y	10.4 ± 1.3	10.6 ± 1.2	0.5	31.3 ± 3.9	31.9 ± 3.8	0.4
10–14, y	10.9 ± 1.4	10.8 ± 1.5	0.5	32.7 ± 4.4	32.6 ± 5	0.6
All girls Age	11.1 ± 1.6	11.1 ± 1.4	0.8	33.4 ± 4.6	33.7 ± 4.2	0.9
5–9, y	10.9 ± 1.6	10.1 ± 1.3	0.7	32.8 ± 4.8	33.8 ± 4.1	0.3
10–14, y	11.3 ± 1.5	11.2 ± 1.5	0.6	33.9 ± 4.4	33.5 ± 4.4	0.3

^1^ The data are based on examination and complete blood count of children of 5 to 14 years old (*n* = 467). Values are mean ± SE. ^2^ Significance between pre- and post-fortification periods (Mann-Whitney U test).

**Table 2:** Red blood cell count and mean corpuscular volume by sex and age in the retrospective study on the children of Ivory Coast: comparison of data between pre-fortification period (2004–2006) and post-fortification period (2008–2010) ^1^.

	Red Blood Cell, 10^6^/mm			Mean Corpuscular Volume, fL		
	Pre	Post	*p*-Value ^2^ (Pre vs. Post)	Pre	Pre	*p*-Value ^2^ (Pre vs. Post)
All subjects	4.3 ± 0.7	4.3 ± 0.6	0.7	76.8 ± 10.3	77.6 ± 7.9	0.02
All boys Age	4.3 ± 0.7	4.2 ± 0.5	0.3	74.9 ± 9.6	76.2 ± 7.9	0.07
5–9, y	4.2 ± 0.7	4.2 ± 0.5	0.4	74.6 ± 10.2	77.2 ± 6.9	0.06
10–14, y	4.3 ± 0.9	4.3 ± 0.5	0.6	75.4 ± 9.2	74.9 ± 8.9	0.7
All girls Age	4.2 ± 0.5	4.4 ± 0.6	0.08	78.7 ± 10.6	79 ± 7.8	0.07
5–9, y	4.4 ± 0.7	4.4 ± 0.6	0.4	75.2 ± 7.8	79.1 ± 8.4	0.01
10–14, y	4.1 ± 0.6	4.3 ± 0.6	0.2	81.8 ± 11.8	78.9 ± 6.9	0.9

^1^ Data are based on examination and complete blood count of children of 5 to 14 years old (*n* = 467). Values are mean ± SE. ^2^ Significance between pre-fortification and post-fortification periods. As the data were not normal, Mann-Whitney U test was used.

**Table 3:** Prevalence of anemia by sex and age in the retrospective study on children of Ivory Coast: comparison of data between pre-fortification period (2004–2006) and post-fortification period (2008–2010) (*n* = 467) ^1^.

	Prevalence of Anemia ^2^				*p*-Value ^3^ (Pre vs. Post)
	Pre		Post		
	Cases, *n*	Prevalence, %	Cases, *n*	Prevalence, %	
All subjects	179	77.2	181	77	0.9
All boys Age	100	82.6	99	80.5	0.7
5–9, y	49	84.5	59	81.9	0.7
10–14, y	51	81	40	78.4	0.7
All girls Age	79	71.2	82	73.2	0.7
5–9, y	40	75.5	45	68.2	0.4
10–14, y	39	67.2	37	80.4	0.1

^1^ Data are based on examination and complete blood count of children of 5 to 14 years old. ^2^ Anemia was defined as having hemoglobin ˂11 g/dL for children under 6 years old and ˂12 g/dL for children 6–14 years old. ^3^ Significance for the prevalence of anemia between pre- and post-fortification periods (chi-squared test).

## Data Availability

Data are available upon request from the authors.
